# Socio-demographic profile of suicide victims in India: A post-mortem analysis

**DOI:** 10.6026/973206300222607

**Published:** 2026-04-30

**Authors:** Vivek Kumar Chouksey, Sudhir Kumar Jain, Brinda Patel, Vaibhav Agrawal

**Affiliations:** 1 Department of Forensic Medicine and Toxicology, Atal Bihari Vajpayee Government Medical College (ABVGMC), Vidisha, Madhya Pradesh, India

**Keywords:** Suicide, socio-economic status, age group, sex

## Abstract

Over 10% of all suicides worldwide occur in India, where majority of cases are young men. Data on suicide victims is scarce, particularly in developing countries like India. Therefore, it is of interest to examine the socio-demographic characteristics of suicide victims in India. Hence, 50 cases with post-mortem examination were studied. Most victims were aged 21-30 years (32%) and had a high education level (38% graduated or post-graduated). A significant number of victims were unemployed (26%) and married (50%), with the majority belonging to lower socio-economic groups (62%). Thus, we document a detailed socio-demographic profile of suicide victims in India, highlighting key factors such as age, education level, employment status and socio-economic background, which can inform targeted suicide prevention strategies.

## Background:

Suicide is generally defined as an intentional move to end one's own life. Suicide has two components; one is ideation followed by suicide plan and both are necessary for ending one's life. Suicidal behavior is expressed as conduct of a person against himself and threatening his own life [1]. Suicide is leading cause of death in western countries like United States of America etc. accounting to almost 2% of the world's total deaths caused by suicides while Asia and India account for more than 60% and 10% of the total suicides in the world [2, 2]. Unfortunately, suicide cannot be predicted. According to the majority of research, between 70 and 95 percent of suicide victims have a curable mental illness, with affective disorders and schizophrenia being the most prevalent. It is well known that most individuals with suicidal tendencies tell those close to them, including their doctors, about their plans to harm themselves [4]. Therefore, with the support of the family and society at large, it is entirely preventable. Diverse racial and ethnic groupings as well as diverse geographic places have an impact on the suicide rates and trends [5]. Culture and society have a significant influence on how people behave when they consider suicide. Therefore, the development and application of suicide prevention strategies are greatly influenced by societal and cultural factors [6]. Although 85% of suicides worldwide take place in low- and middle-income nations, according to data from the WHO mortality database, the majority of what we know and comprehend about suicidal behavior is based on data from high-income nations, which might not be relevant in other cultural contexts [7]. Furthermore, statistics on suicide victims in under developed nations like India are essentially nonexistent. Therefore, it is of interest to report the socio-demographic profile of suicide victims in detail and to develop preventative strategies because, in contrast to suicide, which ends a person's life forever, parasuicide paralyzes a person's life either permanently or temporarily.

## Materials and Methods:

The current study was carried out on 50 suicide cases that were taken to the mortuary for post-mortem investigation at Lady Hardinge Medical College's, Department of Forensic Medicine and Toxicology. After gathering the necessary information from the family members and the accompanying investigating officer, the socio-demographic profile of suicide victims was examined. Following formal written agreement from family members to supply the necessary data for the aforementioned study, the case history was documented on a proforma in accordance with the protocol. A brief background of the incident, personal history, any contributing factors and family history were also included in the proforma. In each case, a psychological autopsy was conducted to try to determine the victim's mental state shortly prior to the incident and the investigating officer provided the information and photos of the crime scene.

## Ethical considerations:

Permission was obtained from the Institutional Ethics Committee prior to conducting the study.

## Statistical analysis:

Data were entered in Microsoft Excel and analyzed using appropriate statistical software SPSS version 24. Descriptive statistics such as frequency, percentage, mean and standard deviation were calculated. A p-value <0.05 was considered statistically significant.

## Results:

Out of a total of fifty cases of suicide, 16 cases (32%) were in 21-30 years age group followed by 10 cases (20%) in 31-40 years bracket Table 1 (see PDF). The males outnumbered the females as maximum number of cases that is 38 (76%) were male and only 11(22%) was female Table 2 (see PDF). Table 3 (see PDF) shows that, maximum number of cases, 19 (38%) managed to complete their graduation level and in sharp contrast just 9 cases (18%) were illiterate. Out of total cases 25, (50%) married persons committed suicide whereas only 18(36%) unmarried persons committed suicide. Least number of suicide incidences was noted in divorced group (2%) Figure 1 (see PDF). Maximum number of cases 13 (26%) were unemployed. 11 (22%) cases belonged to student and manual labour class respectively Table 4 (see PDF). Most of cases 31(62%) belonged to lower class socio-economic status followed by 18 (36%) cases belonged to middle class and least number of cases (2%) belonged to upper class Table 5 (see PDF). Figure 2 (see PDF) shows that 33 (66%) victims had home as their preferred place for commission of suicide. Only 7 (14%) victims used hotel and office as their preferred place whereas the remaining 3 (6%) had committed suicide at other places. Violent methods of suicide such as hanging was used by 31 (62%) persons to commit suicide, 5 (10%) killed themselves by self- immolation whereas 1(2%) person shot himself and 1(2%) fell from height. Non-violent methods were used by 12 (24%) persons who poisoned themselves Table 6 (see PDF).

## Discussion:

Suicide is increasingly recognized as a major public health concern worldwide, with a significant burden observed in low- and middle- income countries such as India. Recent global and national reports continue to highlight a rising trend in suicide rates, particularly among young adults, emphasizing the need for region-specific epidemiological data and preventive strategies [8]. The present study demonstrates that the highest incidence of suicide occurred in the third decade of life, closely followed by the fourth decade, consistent with previous studies reporting the majority of victims in the 21-30 year age group [9]. Globally, suicide remains one of the leading causes of death among young individuals, particularly those aged 15-29 years, with a significant proportion occurring between 15-44 years. National data from India similarly indicate that youth (18-30 years) and middle-aged adults (30-45 years) constitute the largest proportion of suicide cases. Contributing factors in younger populations include academic and peer pressure, parental control, gender-related issues and exposure to abuse [10]. In the present study, males significantly outnumbered females in suicide cases, consistent with previous research demonstrating male predominance with varying male-to-female ratios [11]. Other studies have also reported a rising proportion of male suicides over time, while female suicide rates showed a decline during the same period [12]. However, some research indicates that females attempt suicide more frequently and in certain populations, females slightly outnumber males [13]. The higher incidence among males may be attributed to financial stress, societal expectations and the burden of being the primary earning member of the family. In females, socio-cultural factors such as marital conflicts, domestic violence, dowry-related issues and pressure to remain in abusive marriages contribute significantly to suicide risk. The present study reported that the married people have higher rate of suicide concurs with the studies done by other authors [14, 15]. Various researchers pointed out that the incidence of suicide is more in single people and single people are more likely to commit suicide than married people [16]. In the present study, maximum number of suicide victims managed to complete their graduation and post-graduation level. Our study is in sharp contrast to various studies of completed suicide that had a large number of victims as illiterate. This could possibly be due to increased professional and social pressure. There is a fairly strong association between suicide rate and unemployment. Unemployment may drive up the suicide risk through factors such as poverty, social deprivation, isolation, domestic difficulties and hopelessness. The present study is in sharp contrast to studies by other authors which reported that unemployed persons are at high risk of committing suicide [17]. Housewives take all the stress and are unable to cope up with the never-ending task of taking care of family thus making them prone to suicide. Economic deprivation is directly associated with suicide. In the present study, most of the victims belonged to lower socio-economic strata, in agreement with findings by other researchers that majority of victims or those with high risk of committing suicide are from low socio- economic strata [18]. In present study, most of the victims committed suicide at home; the location of suicide offers clues to the individual's psychological state and to the intentionality of suicide. The present study's results are in agreement with studies done by various authors where home was the most common location of suicide [19]. Recent literature and government data also emphasizes the impact of emerging stressors such as economic uncertainty, mental health burden and societal transitions on suicidal behavior, particularly in the post-pandemic period [20]. These findings underscore the importance of strengthening mental health services, improving early identification of at-risk individuals and implementing targeted suicide prevention strategies.

## Conclusion:

Suicide predominantly affects young adults, especially males, with higher vulnerability among individuals from lower socio-economic backgrounds. Socio-familial stressors, unemployment and educational pressures appear to be key contributing factors. Strengthening mental health awareness and socio-economic support systems is essential for effective suicide prevention.
